# Supplementary material for: A standardised protocol for measuring farmland biodiversity outcomes across European Farmer Cluster landscapes
Source: PLoS One. 2026 Mar 25;21(3):e0345691. doi: 10.1371/journal.pone.0345691 (PMC13016360; doi:10.1371/journal.pone.0345691)
Supplement: S1 Appendix — (DOCX) [file pone.0345691.s001.docx]

**S1 Appendix**

BACI equation requires an ideal output of 4 for the number of impact and control squares to be able to reliably detect an effect (Christie et al., 2019)


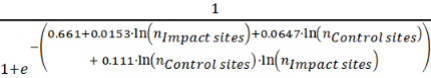


Christie AP, Amano T, Martin PA, Shackelford GE, Simmons BI, Sutherland WJ. Simple study designs in ecology produce inaccurate estimates of biodiversity responses. Journal of Applied Ecology. 2019 Dec 19;56(12):2742–54
