# Supplementary material for: A standardised protocol for measuring farmland biodiversity outcomes across European Farmer Cluster landscapes
Source: PLoS One. 2026 Mar 25;21(3):e0345691. doi: 10.1371/journal.pone.0345691 (PMC13016360; doi:10.1371/journal.pone.0345691)
Supplement: S2 Appendix — (DOCX) [file pone.0345691.s002.docx]

**S2 Appendix. Full list of potential biodiversity indicators and survey difficulty level calculation method**

| **Species diversity indicators** | | | **Present in BioBio** | **National survey guidelines** | **Landscape scale** | **Suggested survey method** | **Difficulty^a^** | **No. partners interested** | **No. partners skilled** |
| --- | --- | --- | --- | --- | --- | --- | --- | --- | --- |
| **Category** | **Primary functional trait** | **Indicator (potential ID level)** |  |  |  |  |  |  |  |
| **Vascular plants** | Primary producer | Flowering plants - crops (species) | x | x |  | Transect/quadrat | 1 x 2 = 2 | 6 | 2 |
|  | Primary producer | Vegetation - species and structure (species) | x | x | x | Transect/quadrat | 1 x 2 = 2 | 6 | 2 |
| **Invertebrates** | Decomposers | Earthworms (species) | x | x |  | Soil pit | 1 x 3 = 3 | 3 | 1 |
|  | Natural enemy | Spiders (species) | x |  |  | Pit fall trap | 3 x 4 = 12 | 4 | 2 |
|  | Pollinator | Butterflies (species) |  | x | x | Transect | 2 x 2 = 4 | 7 | 3 |
|  | Pollinator/pest | Moths (day flying) (Macromoths to species) |  |  |  | Transect | 2 x 2 = 4 | 4 | 1 |
|  | Pollinator/pest | Moths (night flying) (Macromoths to Genus) |  | x | x | Light trap | 3 x 2 = 6 | 4 | 1 |
|  | Pollinator | Bumblebees (species or morphospecies) | x | x | x | Transect | 2 x 2 = 4 | 8 | 4 |
|  | Pollinator | Solitary bees (species) | x |  |  | Pan-trap | 3 x 4 = 12 | 8 | 4 |
|  | Other | Ants (Family) |  |  |  | Pit fall trap | 3 x 4 = 12 | 0 | 0 |
|  | Natural enemy | Wasps (predators) (species) |  |  | x | Transect/pan trap | 3 x 2 = 6 | 0 | 0 |
|  | Natural enemy | Parasitoids (Family/Genus) |  |  |  | Pan trap | 3 x 4 = 12 | 0 | 0 |
|  | Herbivore | Snails and slugs (species) |  |  |  | Baited trap | 3 x 2 = 6 | 1 | 0 |
|  | Natural enemy/herbivore | Carabid beetles (species) |  | x |  | Pitfall trap | 3 x 4 = 12 | 5 | 2 |
|  | Pollinator | Hoverflies (species) |  |  | x | Transect/pan trap | 3 x 4 = 12 | 6 | 3 |
|  | Predator | Syrphid larvae (Genus/species) |  |  |  | Transect | 2 x 4 = 8 |  |  |
|  | Pest | Aphidoidea (species) |  |  |  | Sweep net (along transect) | 2 x 4 = 8 | 2 | 0 |
|  | Natural enemy | Staphylinidae (species) |  |  |  | Vacuum sampling | 2 x 4 = 8 | 1 | 1 |
|  | Aquatic | Odonata (species) |  |  |  | Net (pond) | 1 x 2 = 2 | 1 | 1 |
|  | Herbivore | Orthoptera (species) |  |  |  | Transect (acoustic device) | 2 x 2 = 4 | 0 | 0 |
|  | Herbivore | Heteroptera (Genus) |  |  |  | Vacuum sampling | 1 x 4 = 4 | 1 | 1 |
|  | Herbivore | Auchenorrhyncha (species) |  |  |  | Vacuum sampling | 1 x 4 = 4 | 1 | 1 |
|  | Decomposers | Collembola (Genus) |  |  |  | Vacuum sampling | 1 x 4 = 4 | 1 | 1 |
|  | Other | Soil Biological Quality – microarthropods) (Taxa) |  |  |  | Burlese funnel trap | 2 x 4 = 8 | 0 | 0 |
| **Vertebrates** | Natural enemy | Amphibians (Genus/species) |  |  | x | Net (pond) | 1 x 2 = 2 | 1 | 0 |
|  | Natural enemy | Reptiles (species) |  |  | x | Artificial refugia | 2 x 2 = 4 | 1 | 0 |
|  | Natural enemy/herbivore | Birds (species) |  | x | x | Transect | 1 x 1 = 1 | 6 | 2 |
|  | Natural enemy | Small mammals (e.g., shrews, rodents) (licences required) |  |  | x | Baited trap | 2 x 2 = 4 | 0 | 1 |
|  | Natural enemy | Bats (species) |  | x | x | Transect (acoustic recording device) | 1 x 3 = 3 | 0 | 0 |
|  | Natural enemy | Carnivores (mammals - Order Carnivora) (species) |  |  | x | Camera trap | 2 x 3 = 6 | 1 | 0 |
|  | Herbivore | Herbivores (mammals - Ungulates, Lagomorphs) (species) |  |  | x | Camera trap | 2 x 3 = 6 | 2 | 1 |
| **Habitat diversity indicators** |  | Habitat richness (N° of habitat types per hectare) | x |  | x | mapping |  |  |  |
|  |  | Habitat diversity (Shannon diversity) | x |  | x | mapping |  |  |  |
|  |  | Average size of habitat patches | x |  | x | mapping |  |  |  |
|  |  | Length of linear elements (m / ha) | x |  | x | mapping |  |  |  |
|  |  | Crop richness (N° of crops per farm / per hectare) | x |  | x | mapping |  |  |  |
|  |  | Percentage of farmland with shrubs (% of farmland) | x |  | x | mapping |  |  |  |
|  |  | Tree habitats (Tree cover % of farmland) | x |  | x | mapping |  |  |  |
|  |  | Percentage of semi-natural habitats (% of farmland) | x |  | x | mapping |  |  |  |
| **Farm management indicators** |  | Total direct and indirect energy input | x |  |  | interview/questionnaire |  |  |  |
|  |  | Intensification/Extensification | x |  |  | interview/questionnaire |  |  |  |
|  |  | Area with use of mineral N-fertiliser | x |  |  | interview/questionnaire |  |  |  |
|  |  | Total nitrogen input | x |  |  | interview/questionnaire |  |  |  |
|  |  | Field operations | x |  |  | interview/questionnaire |  |  |  |
|  |  | Pesticide use | x |  |  | interview/questionnaire |  |  |  |
|  |  | Average stocking rate | x |  |  | interview/questionnaire |  |  |  |
|  |  | Grazing intensity | x |  |  | interview/questionnaire |  |  |  |
| **Genetic diversity of livestock** |  | Number of different breeds | x |  |  | interview/questionnaire |  |  |  |
|  |  | Number of different varieties | x |  |  | interview/questionnaire |  |  |  |
|  |  | Origin of crops | x |  |  | interview/questionnaire |  |  |  |

Indicator list formulated through project partner collaboration and discussion over multiple sessions. Indicators highlighted (green rows) were selected for biodiversity monitoring. The species diversity indicators are split into categories with their primary functional traits listed. The potential/suggested identification level is included in brackets. It is noted (x) if the taxa is present in BioBio (a European-wide farmland biodiversity study); if national survey guidelines for surveying the taxa in any European countries exist; and if the taxa is known to be indicative of activity at the landscape-scale. The suggested survey methods are included. The difficulty level in collecting the data is calculated by multiplying the survey effort by the species ID effort, and falls between 1 (minimum) and 12 (maximum). Also included are the number of project partners interested in collecting data on the taxa, and the number of project partners skilled in identifying species within the taxa.

^a^Difficulty in completing surveys and identifying to appropriate species level (survey effort x species ID effort): ranges from 1 (minimum) to 12 (maximum) (see below).

Difficulty calculations:

|  |  |  | Survey effort | |  |  |  |  |
| --- | --- | --- | --- | --- | --- | --- | --- | --- |
|  | simple | 1 | 2 | 3 | 4 |  |  |  |
|  | 1 | 1 | 2 | 3 | 4 |  |  |  |
| ID effort | 2 | 2 | 4 | 6 | 8 |  |  |  |
|  | 3 | 3 | 6 | 9 | 12 |  |  |  |
|  | 4 | 4 | 8 | 12 | 16 |  |  |  |
|  |  |  |  |  |  | complex |  |  |
|  | **Survey effort per annum** | | |  |  |  |  |  |
|  | 1 | 1 to 5 visits | |  |  |  |  |  |
|  | 2 | 6 to 10 visits | |  |  |  |  |  |
|  | 3 | 11 to 15 visits | |  |  |  |  |  |
|  | 4 | >15 visits |  |  |  |  |  |  |
|  |  |  |  |  |  |  |  |  |
|  | **ID effort** |  |  |  |  |  |  |  |
|  | 1 | in-field during survey - timed walk | | | |  |  |  |
|  | 2 | in-field during survey | | |  |  |  |  |
|  | 3 | returned to lab, preserved samples, simple key (visual) | | | | | |  |
|  | 4 | returned to lab, preserved samples, technical key (microscope) | | | | | | |
|  |  |  |  |  |  |  |  |  |
