# Supplementary material for: A standardised protocol for measuring farmland biodiversity outcomes across European Farmer Cluster landscapes
Source: PLoS One. 2026 Mar 25;21(3):e0345691. doi: 10.1371/journal.pone.0345691 (PMC13016360; doi:10.1371/journal.pone.0345691)
Supplement: S3 Appendix — (DOCX) [file pone.0345691.s003.docx]

**FRAMEwork breeding bird survey field form**

| Observer name: |  |  |  |  |
| --- | --- | --- | --- | --- |
| Treatment: |  |  | Survey Square: |  |
| Survey round: | ONE TWO |  | Date: | **/ /** |
| Start time: |  |  | Sunrise time: |  |

Weather (tick the appropriate categories):

| **Cloud** |  | **Rain** |  | **Wind** |  | **Visibility** |  |
| --- | --- | --- | --- | --- | --- | --- | --- |
| 0-33% |  | None |  | Calm |  | Good |  |
| 33-66% |  | Drizzle |  | Light |  | Moderate |  |
| 66-100% |  | Showers |  | Breezy |  | Poor |  |

| SECTION: | | | |  | | | | | | | |  | SECTION: | | | |  | | | | | | |
| --- | --- | --- | --- | --- | --- | --- | --- | --- | --- | --- | --- | --- | --- | --- | --- | --- | --- | --- | --- | --- | --- | --- | --- |
| Start time: | | | |  | | Finish time: | | | |  | |  | Start time: | | | |  | | Finish time: | | | |  |
|  | 100 | | 25 | | 0 | | 25 | | 100 | |  |  |  | 100 | | 25 | | 0 | | 25 | | 100 | |
|  | |  | |  | |  | |  | |  | |  |  | |  | |  | |  | |  | |  |
| In flight only: | | | | | | | | | | | |  | In flight only: | | | | | | | | | | |

**Breeding Bird Codes – adapted from information on the BTO website (bto.org)**

Observations of bird behaviour can be reliably used to infer the breeding status of many species. In each square keep a look out for the behaviours listed below that are indicative of breeding and record them on your data recording sheet beside the relevant observation. For example  CH - CF for a Chaffinch carrying food in its beak or WP- C for a displaying Woodpigeon.

The behaviour codes below are listed in order of increasing certainty of breeding. Please record the highest breeding code observed.

**PROBABLE**

**P – Pair in Suitable Habitat**: Pair observed in suitable nesting habitat during the breeding season. Only use this code when you are fairly certain that a mated pair of birds has been observed. Look for behavioural cues to determine if you have a male-female pair, particularly in species where males and females look the same.

**T – Territorial Defence:** Permanent territory presumed through defence of breeding territory by fighting or chasing. While this is generally used for individuals of the same species, an interaction between members of different species may fall under this code when it appears to be territorial defence. Also see “A – Agitated Behaviour.”

**C – Courtship, Display, or Copulation**: Courtship behaviour or copulation between a male and a female. Courtship behaviour includes transfer of food, displays, and grooming between a pair of birds.

**N – Visiting Probable Nest Site:** Repeated visits to a probable nest site. This is especially useful for cavity nesters or for a shrub-nesting species that flies into the same thicket and disappears on several occasions.

**A – Agitated Behaviour:** Agitated behaviour or anxiety calls from adults indicating a nest site or young in the vicinity. This code refers to a stronger reaction to intruders than those exhibited by “T – Territorial Defence,” usually against brood parasites, nest predators, and humans. This code also excludes mobbing behaviour that species engage in year-round (e.g., mobbing a buzzard).

**CONFIRMED**

**CN – Carrying Nesting Material:** Adult carrying nesting material to an unseen nest, such as sticks, grass, mud, and cobwebs. For raptors, be sure the material is not simply incidental to prey capture/transport.

**NB – Nest Building:** Nest-building observed at the actual nest site.

**DD – Distraction Display:** Distraction displays and injury feigning in attempt to draw intruder away from nest or young.

**UN – Used Nest:** Used nest found, but no adult birds seen nearby. Use only if the nest was used during the current breeding season and you are certain of the species. Add comments detailing how you identified the nest. Do not collect the nest, but do take a photograph if possible.

**ON – Occupied Nest:** Occupied nest indicated by adult sitting in nest in incubating position, adult entering nest site and remaining, or exchange of incubation duties by the pair. This code is useful for nests high in trees and in chimneys where the contents of the nest and incubating or brooding adult cannot be seen.

**FL – Recently Fledged Young**: Recently fledged or downy young still dependent upon adults and presumed incapable of extended flights from nest site. Look for retained downy feathers, a yellow gape, a short tail (shorter than the wings), clumsy flight and landings, and a bird incapable of feeding itself. Beware of family groups late in the breeding season which may still be interacting but are far from the breeding location. If you find a dead fledgling and don’t see an adult of the same species, use the code FL (dead).

**CF – Carrying Food:** Adult carrying food for young or incubating partner. This code should not be used for corvids, raptors, terns, and other species that regularly carry food for courtship, caching, or other purposes. One of the best signs to look for is the repeated carrying of food in the same direction.

**FY – Feeding Young:** Adult bird feeding recently fledged young that are not yet flying and independent. This code should not be used for species that may move many miles from the nest site, such as raptors and terns. Use the NY code for nestlings being fed by an adult.

**FS – Carrying Faecal Sac:** Adult carrying faecal sac or egg shell fragments. Many passerine adults keep their nests clean by carrying membranous, white faecal sacs and broken eggshells away from the nest. Note that only songbirds and woodpeckers produce faecal sacs and this code should only be applied to these groups of species.

**NE – Nest with Eggs:** Nest with eggs. Be careful not to disturb the vicinity of the nest. Confirm the species by waiting at a distance until the adult returns. If no birds are seen, use the UN code.

**NY – Nest with Young**: Nest with young seen or heard. Keep your distance so nestlings are not prematurely flushed from the nest.

**Record how all birds were detected**. The categories are Song, Call, Visual, Song + Visual and Call + Visual. Record the way you first detected the bird (or group). If a bird is detected by sight, but later starts to sing, record this as a Song + Visual detection.

- - Record your data on your field sheets by circling a singing bird, underlining a calling bird, or leaving a record unmarked if you detected a bird visually. If a bird is recorded calling or singing bird is also seen add (V) to the code for visual detection.
  - Flushed birds (detected by sound of wings flapping rather than call) should be indicated by adding the word (flush) after the species code. This mostly applies to Woodpigeons and gamebirds.
