# Supplementary material for: A standardised protocol for measuring farmland biodiversity outcomes across European Farmer Cluster landscapes
Source: PLoS One. 2026 Mar 25;21(3):e0345691. doi: 10.1371/journal.pone.0345691 (PMC13016360; doi:10.1371/journal.pone.0345691)
Supplement: S4 Appendix — (DOCX) [file pone.0345691.s004.docx]

**S4 Appendix**

**An illustration of how the total number of transects might be distributed between broad habitats in each survey square under two extreme cluster configurations.**

| Cluster configuration | Number of squares to survey | BHCs per square | Transects per BHC per square | Transects per square | Total transects |
| --- | --- | --- | --- | --- | --- |
| Small and uniform cluster | 8 squares: 4 cluster + 4 control | 3 | 4 | 12 | 96 |
| 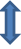 |  | | | | |
| Large diverse cluster | 16 squares: 8 cluster + 8 control | 6 | 1 | 6 | 96 |

**An illustration of how the total number of pan-traps might be distributed between habitats in each survey square under two extreme cluster configurations.**

| Cluster configuration | Number of squares to survey | BHCs per square | Pan-traps per BHC per square | Pan-traps per square | Total pan-traps |
| --- | --- | --- | --- | --- | --- |
| Small and uniform cluster | 8 squares: 4 cluster + 4 control | 3 | 2 | 6 | 48 |
|  |  | | | | |
| Large diverse cluster | 16 squares: 8 cluster + 8 control | 6 | 1/2 | 3 | 48 |
