# Supplementary figures and images for: A standardised protocol for measuring farmland biodiversity outcomes across European Farmer Cluster landscapes
Source: PLoS One. 2026 Mar 25;21(3):e0345691. doi: 10.1371/journal.pone.0345691 (PMC13016360; doi:10.1371/journal.pone.0345691)

**FRAMEwork pollinators transect survey sheet**


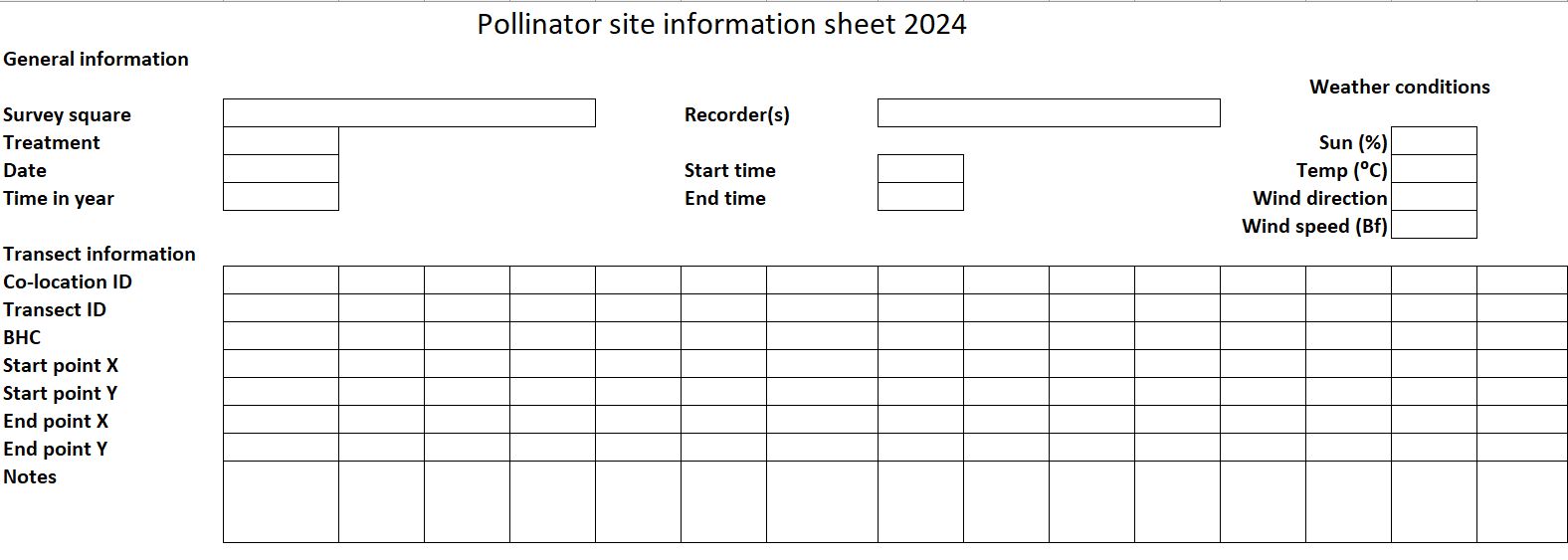

Supplement: S5 Appendix — (DOCX) [file pone.0345691.s005.docx]
