# Supplementary material for: A standardised protocol for measuring farmland biodiversity outcomes across European Farmer Cluster landscapes
Source: PLoS One. 2026 Mar 25;21(3):e0345691. doi: 10.1371/journal.pone.0345691 (PMC13016360; doi:10.1371/journal.pone.0345691)
Supplement: S6 Appendix — (DOCX) [file pone.0345691.s006.docx]

**S6 Appendix**

**Details on pan traps**

Pan-traps consist of small plastic bowls painted with UV reflective paint to form a set of three colours (white, UV blue, and UV yellow) attractive for pollinators, containing water and a drop of (unscented) liquid soap (to break surface tension) in which insects land and drown. They are set on stakes **at vegetation height** to mimic flowers and collected after a pre-specified duration. Pan-traps particularly target small solitary bees (especially Halictidae) and hoverfly species, but also catch non-target insects such as flies, wasps and beetles. Other pollinators tend to be under-sampled, such as *Colletes* spp. and larger bees (e.g. bumblebees) that can escape more easily.

**How to paint the pan-traps**

You will need a primer and an acrylic or glycerol paint. **/!\** Wear protective gear: a mask, gloves, security glasses.

Steps:

1. Lightly rub the surface of the bowl with 220 or 300 grit sandpaper. Press lightly and move in a circular motion.
2. Rinse or wipe the surface.

| 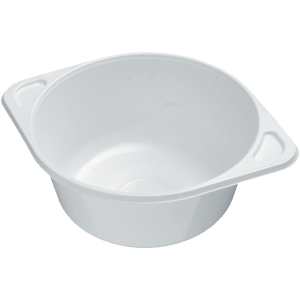 | 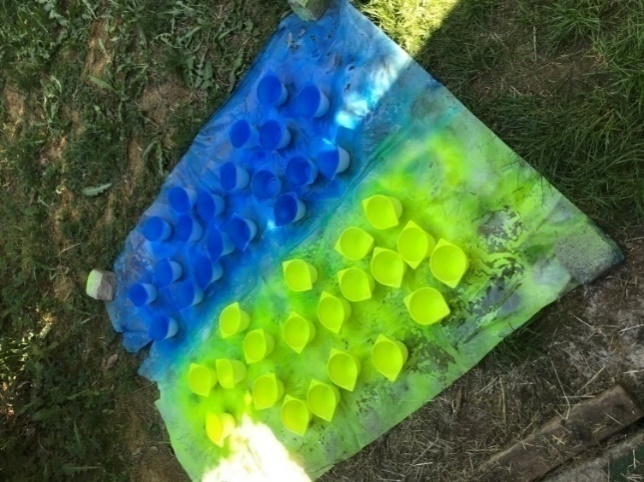 |
| --- | --- |
| *Examples bowls used for the pan-traps.* | |

1. To ensure good adhesion of the paint, first spray a thin layer of primer (e.g. MOTIP brand; “Universal Grundierfarbe”) on the bowl, avoiding accumulations (no need to put too much). Keep the spray at a constant distance of 25 cm from the surface during spraying. Two thin layers of primer are recommended. Let dry between layers (15-20 min).
2. Spray a **thin** layer of the **UV** colour paint in the same way as the fixer. Allow drying before adding a second layer. It is better to put 2-3 thin layers than one thick layer.

- Allow each coat of paint to dry before applying the next one (at least 15 minutes)
- Change the direction of the spraying for each layer

The fluorescent paint should be applied on a white (or transparent) bowl for maximum luminosity. When exposed to UV, the colour tends to fade, the extent of this phenomenon being directly related to the thickness of the paint and the intensity of the UV rays. The blue colour has a less good “longevity” (it sometimes “flakes”, due to the nature of the pigment), so you may need to spray 3-4 layers for this colour.

Some **examples** of spray-paint brands used for pan-traps:

- A brand widely used is SparVAR (<http://www.spraycolor.de/>; RAL Leuchtfarbe):
- Blue = fluorescent blue 3107 LF
- Yellow = fluorescent yellow 3104
- White = fluorescent white 3108

(e.g., <https://doi.org/10.1890/07-1292.1>, <http://dx.doi.org/10.1016/j.biocon.2017.08.027>, <https://doi.org/10.1007/s00442-015-3255-0>, <http://dx.doi.org/10.1016/j.agee.2014.08.016>, https://doi.org/10.1111/2041-210X.13292)

- Paints used by the University of Mons (Belgium) are from Manutan S.A. (Bruxelles, <https://www.manutan.be/fr/mab/peinture-fluorescente-en-aerosol#descriptionAnchor>):
  - Blue = “traceur de chantier Top automatique bleu réf A013273 »
  - Yellow = « traceur de chantier Top automatique jaune réf A013269 »
  - White = « traceur de chantier Top automatique blanc réf A013268 »

(e.g., DOI: 10.3390/insects10020040)

Other examples of pan-trap sets can be found here:

- <https://www.stanleyecologylab.org/>
- <https://oneecosystem.pensoft.net/article/14014/>
- <https://www.mdpi.com/2075-4450/7/4/62>
