# Supplementary material for: A standardised protocol for measuring farmland biodiversity outcomes across European Farmer Cluster landscapes
Source: PLoS One. 2026 Mar 25;21(3):e0345691. doi: 10.1371/journal.pone.0345691 (PMC13016360; doi:10.1371/journal.pone.0345691)
Supplement: S7 Appendix — (DOCX) [file pone.0345691.s007.docx]

**FRAMEwork pan-trap survey sheet**
