# Supplementary material for: A standardised protocol for measuring farmland biodiversity outcomes across European Farmer Cluster landscapes
Source: PLoS One. 2026 Mar 25;21(3):e0345691. doi: 10.1371/journal.pone.0345691 (PMC13016360; doi:10.1371/journal.pone.0345691)

**S8 Appendix**

**Flower unit examples and descriptions.**

*(Source: UK Pollinator Monitoring Scheme)*

Species indicators taken from EMBAL Survey Manual 2021.

Indictaor species for **arable land**


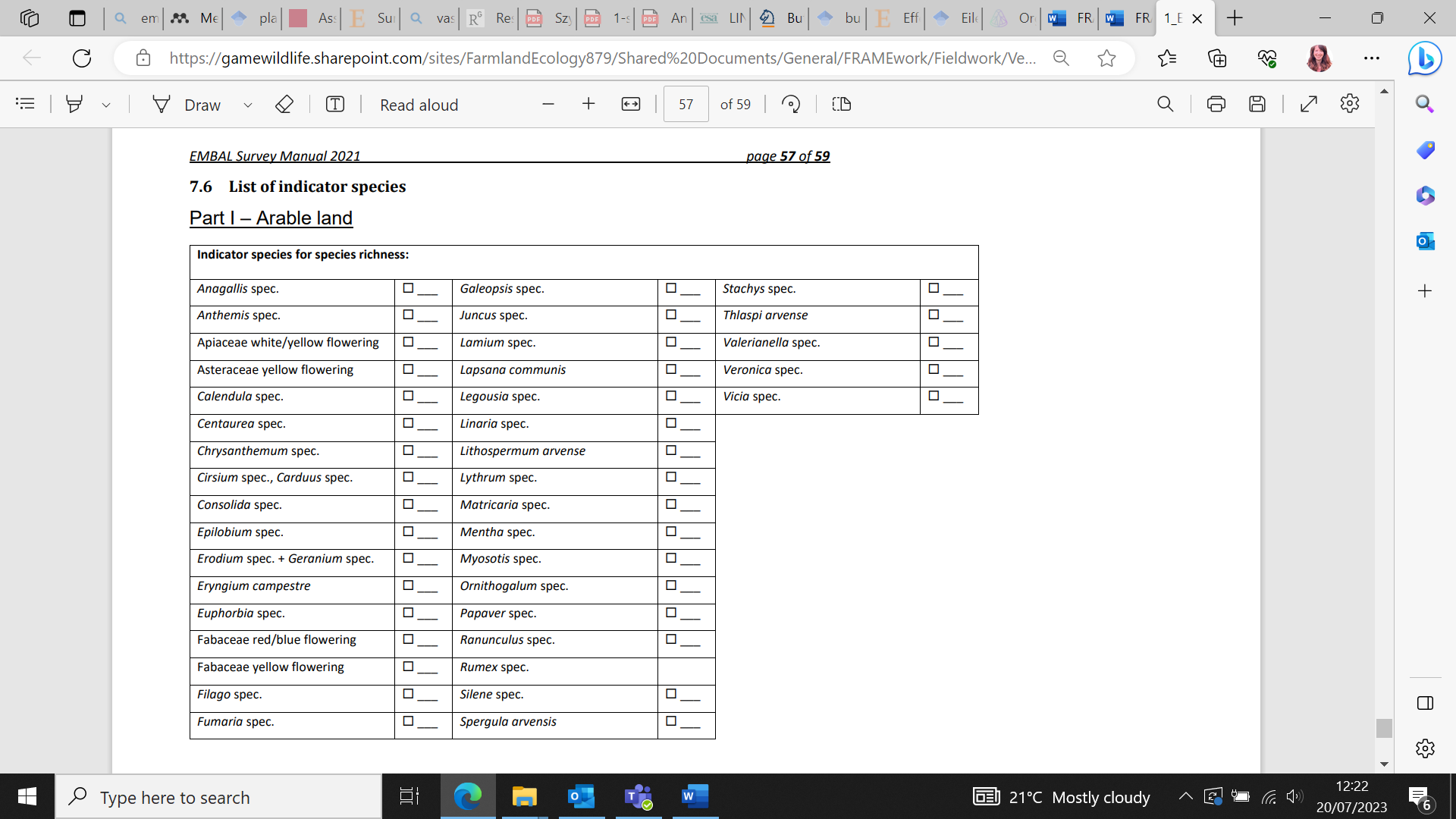


Indicator species for **grassland**


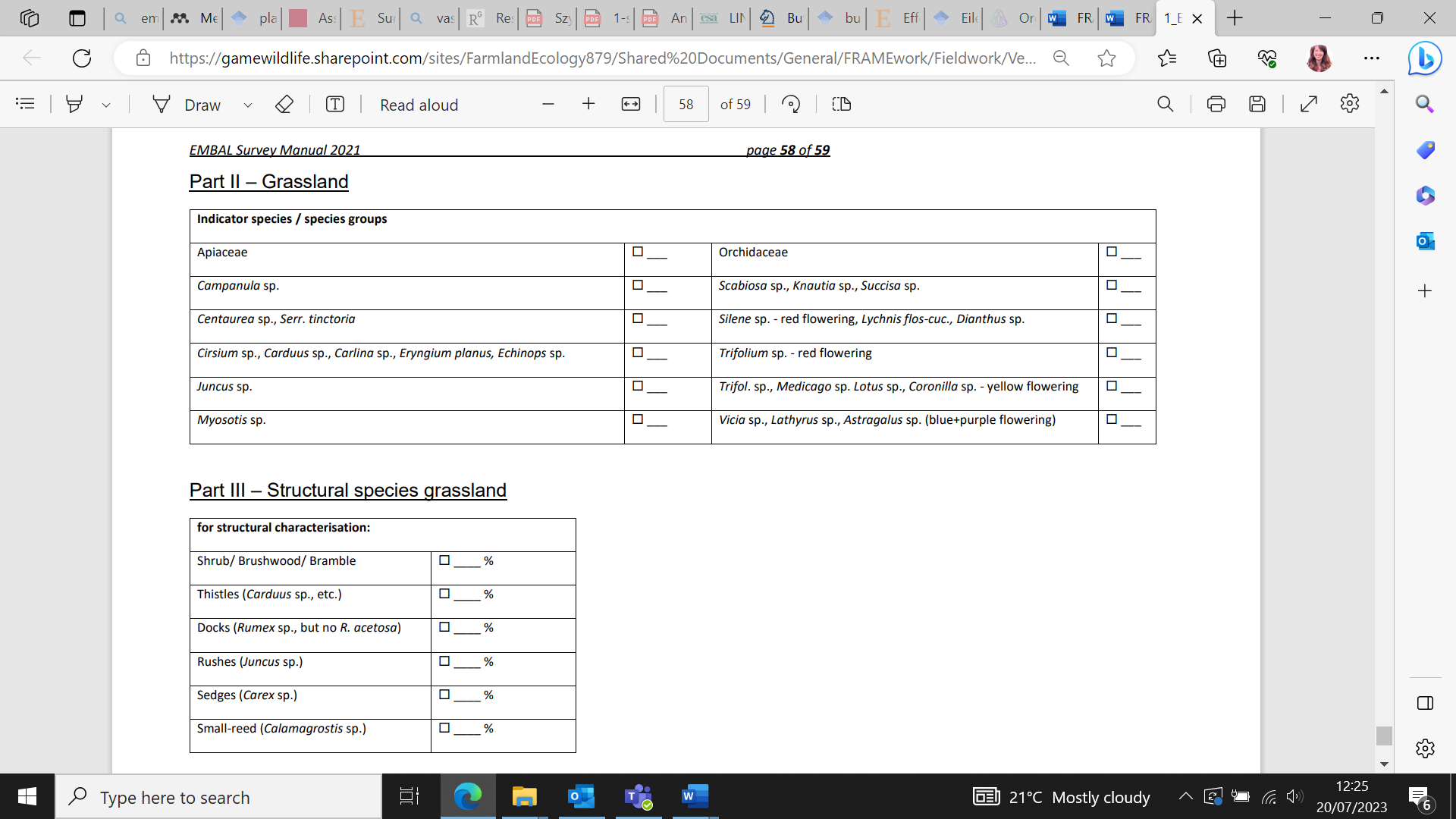

Supplement: S8 Appendix — (DOCX) [file pone.0345691.s008.docx]
