# Supplementary material for: A standardised protocol for measuring farmland biodiversity outcomes across European Farmer Cluster landscapes
Source: PLoS One. 2026 Mar 25;21(3):e0345691. doi: 10.1371/journal.pone.0345691 (PMC13016360; doi:10.1371/journal.pone.0345691)
Supplement: Attachment — Submitted filename: response to reviewers.docx [file pone.0345691.s010.docx]

PONE-D-24-21560

The Farmer Cluster approach for effective agroecology: a standardised protocol for measuring farmland biodiversity outcomes at the landscape-scale across Europe

PLOS ONE

Dear Dr. Nichols,

Thank you for submitting your manuscript to PLOS ONE. After careful consideration, we have decided that your manuscript does not meet our criteria for publication and must therefore be rejected.

Specifically:

Dear Rachel Naomi Nichols,

your manuscript has been reviewed by two referees who both identified serious and complementary concerns with this manuscript in its present form. I thus cannot accept it for publication. I also doubt that a revision would enable to solve the issues raised by the reviewers, without completely reshaping this manuscript. I must say that the reviewers have made a very detailed and thoughtful appraisal of your work. They make numerous constructive comments that should help you strengthen your manuscript.

I am sorry that we cannot be more positive on this occasion, but hope that you appreciate the reasons for this decision.

Kind regards,

Franck Jabot

Academic Editor

PLOS ONE

Additional Editor Comments:

Dear Rachel Naomi Nichols,

your manuscript has been reviewed by two referees who both identified serious and complementary concerns with this manuscript in its present form. I thus cannot accept it for publication. I also doubt that a revision would enable to solve the issues raised by the reviewers, without completely reshaping this manuscript. I must say that the reviewers have made a very detailed and thoughtful appraisal of your work. They make numerous constructive comments that should help you strengthen your manuscript for a future submission.

Kind regards,

Franck Jabot

[Note: HTML markup is below. Please do not edit.]

Reviewers' comments:

Reviewer's Responses to Questions

Comments to the Author

1. Is the manuscript technically sound, and do the data support the conclusions?

The manuscript must describe a technically sound piece of scientific research with data that supports the conclusions. Experiments must have been conducted rigorously, with appropriate controls, replication, and sample sizes. The conclusions must be drawn appropriately based on the data presented.

Reviewer #1: Partly

Reviewer #2: Yes

________________________________________

2. Has the statistical analysis been performed appropriately and rigorously?

Reviewer #1: I Don't Know

Reviewer #2: N/A

________________________________________

3. Have the authors made all data underlying the findings in their manuscript fully available?

The PLOS Data policy requires authors to make all data underlying the findings described in their manuscript fully available without restriction, with rare exception (please refer to the Data Availability Statement in the manuscript PDF file). The data should be provided as part of the manuscript or its supporting information, or deposited to a public repository. For example, in addition to summary statistics, the data points behind means, medians and variance measures should be available. If there are restrictions on publicly sharing data—e.g. participant privacy or use of data from a third party—those must be specified.

Reviewer #1: Yes

Reviewer #2: Yes

________________________________________

4. Is the manuscript presented in an intelligible fashion and written in standard English?

PLOS ONE does not copyedit accepted manuscripts, so the language in submitted articles must be clear, correct, and unambiguous. Any typographical or grammatical errors should be corrected at revision, so please note any specific errors here.

Reviewer #1: Yes

Reviewer #2: Yes

________________________________________

5. Review Comments to the Author

Please use the space provided to explain your answers to the questions above. You may also include additional comments for the author, including concerns about dual publication, research ethics, or publication ethics. (Please upload your review as an attachment if it exceeds 20,000 characters)

Reviewer #1:

Nichels et al. presented in their study, entitled The Farmer Cluster approach for effective agroecology: a standardized protocol for measuring farmland biodiversity outcomes at the landscape-scale across Europe, parts of the FRAMEwork project.

The overall objectives of the study were

(i) to describe the different strategies used to established Farm Clusters (FC), (ii) to develop and apply a standardized protocol for surveying farmland biodiversity at the landscape-scale (iii) based on identifyed biodiversity indicators.

This approach is complemented by a hypothetical case study to demonstrate how the methods produce results that can be amalgamated, contributing towards a multi-taxa, community-level, landscape-scale biodiversity analysis.

General comments

In Europe, almost 40 % of the land is used for agriculture. This means that agriculture plays an important role in conserving and promoting biodiversity. The topic of this study is highly relevant, particularly considering the current climate, biodiversity and food crises.

Unfortunately, the study has considerable weaknesses in terms of content and structure, so that numerous questions arise when reading the manuscript and the flow of reading is often interrupted as a result. I strongly recommend sharpening the introduction and ending it with clearly formulated questions. These can then serve as a guide for the manuscript and helps to give it more structure. To emphasize the singularity of the study, the methods and results section need to be revised or complemented. It would be desirable to discuss the results in the light of existing approaches and to put them in the agricultural policy context.

**>> The manuscript has been greatly improved in its aims and content. The introduction now clearly states the aims of the paper. We have a clear anticipated results section (as this is still a methods paper), and a discussion that assesses the methods used.**

In the following, I would like to share my thoughts and questions on the individual parts of the manuscript with the authors:

Introduction

The introduction begins in a clearly structured and well-formulated way and makes you want to read the manuscript. This is particularly true for the first part of the introduction (AES, FC in the UK). Although a large part of the manuscript is dedicated to biodiversity monitoring and this is one of the main focuses of the study, the actual challenges in this context are not addressed: How can one a) estimate possible effects of biodiversity measures at a landscape-scale (in the sense of statistically validated results) and b) distinguish these from possible confounding landscape effects (e.g. the presence of valuable landscape structures on the land of FC, effects of former measures, land use history)?

**>> We thank the reviewer for their comments. We understand the reviewer’s concerns regarding former land management; however, we feel this was outside the scope of this paper. Please find the below sections of text within the manuscript that capture a response to your other concerns:**

**L119-126: “Therefore, to determine the effect of FCs on overall farmland biodiversity, a BACI experiment was designed. Each FC was paired with a comparable control in a nearby, similar landscape and farming system (approximately 2 km away; Fig 1B). This approach allows the assessment of change in biodiversity over time by comparing trends in intervention and control sites before and after FC biodiversity measures were implemented. Before the project commenced, a formula (19; S1 Appendix) was used to calculate that a minimum of four treatment (FC) squares and four control squares were required to be 72.8% confident of correctly predicting the direction of biodiversity change on control versus FC site by the end of the project in a BACI analysis.”**

**L400-404: “In terms of the BACI analysis, if the null hypothesis is rejected, the FCs will show an increase in biodiversity over time in comparison to the control sites (illustrated by Fig 4A). Landscape descriptors (e.g. SNH coverage, habitat fragmentation) should also be statistically compared between the FC and control squares to adjust for any confounding impacts.”**

It is surprising that the authors claim that there are no multi-taxa assessments of farmland biodiversity at landscape scale. In Switzerland, for example, this is done as part of the ALL-EMA farmland biodiversity monitoring programme (https://www.agroscope.admin.ch/agroscope/en/home/topics/environment-resources/monitoring-analytics/monitoring-programm-all-ema.html) and in the Austrian monitoring of cultural landscapes (ÖBM Kulturlandschaften). There are also numerous examples of living labs that pursue multi-taxa approaches.

**>> We apologise for this oversight in the text. The text now reads:**

**L87-89: Although several large multi-taxa programmes exist with well-developed protocols aimed at monitoring biodiversity (Herzog et al., 2022; Staley et al., 2022; Opperman et al., 2021), none were designed specifically to operate at the scale of the FC or the diverse landscape sizes that they encompass.**

What is the methodological novelty compared to other studies? In the stratified survey approach?

**>> L87-91: Although several large multi-taxa programmes exist with well-developed protocols aimed at monitoring biodiversity (Herzog et al., 2022; Staley et al., 2022; Opperman et al., 2021), none were designed specifically to operate at the scale of the FC or the diverse landscape sizes that they encompass. There was a need to identify indicator species that were relevant to the landscape and aligned with the interests of the FCs, as well as a protocol adaptable across different European countries and farming systems.**

What are the specific questions that the study aims to answer?

**>> L100-104: The aim of this paper is to a) describe the FRAMEwork project’s methods to select and survey landscape-scale biodiversity indicators, and b) evaluate the appropriateness of the selected taxonomic indicators and the methodologies for data collection. However, the described protocols are also intended to be applicable for any future FC facilitators or practitioners to monitor their FC biodiversity from inception.**

What was your motivation to include the aspect of forming FC in the manuscript? In my opinion this aspect is not relevant for the development of the monitoring approach. To leave it out would make the manuscript more straight forward.

**>> We agree with the reviewer. This has now been removed from the manuscript. This paper now only deals with the biodiversity elements of the study.**

Methods

FRAMEwork locations

It would be useful for readers who are not familiar with the project to provide some background information about FRAMEwork.

**>> L92-98: The FRAMEwork project (Farmer clusters for Realising Agrobiodiversity Management across Ecosystems), a multi-partner project funded by the EU Horizon 2020 initiative, was established to investigate whether the FC approach could be employed across Europe. A key aspect of FRAMEwork was the development and testing of methods and tools for the monitoring and evaluation of farmland biodiversity, a main criterion for assessing the performance of the FCs. The overarching aim was to determine if biodiversity could be improved overall across the landscape by the FC selecting their own biodiversity targets and biodiversity-friendly farming initiatives.**

Formation of Farmer Clusters

l. 154: It is important for the understanding of the reader to know what farmer-led biodiversity objectives are. It would be good to give examples and categorise them if necessary. It would also be helpful to indicate whether these objectives are within the scope of legal requirements or are significantly more ambitious. What motivated farmers to increase biodiversity? What measures have farmers implemented to tackle these objectives? This information would be important at this point in the manuscript.

l. 160: What are the demands and constraints of the project?

l. 162: ‘in some cases with specific landscape-scale conservation aims’ – This contradicts the farmer-led biodiversity objectives. Please explain.

**>> All of the above text regarding forming a farmer cluster has been removed from the paper. This paper now only deals with the biodiversity elements of the study.**

Biodiversity monitoring

This part raised many questions for me.

The design of any monitoring programme is determined by the underlying questions and objectives. I think this is where the weakness of the manuscript becomes clear: what are the specific questions that the study is intended to answer?

Perhaps the questions could be formulated as follows:

• Do the AES implemented by FC have a positive impact on biodiversity?

• How should biodiversity monitoring be designed so that it can distinguish possible effects of biodiversity measures from confounding landscape effects at the FC level?

• Which groups of organisms are suitable as indicators? (depending on the motivation of the FC  functional diversity (pollination, pest control, etc.) and/or species conservation)

At this point, I would like to recommend the paper by Reynolds et al. (2016) - A road map for designing and implementing a biological monitoring' (Environmental Monitoring and Assessment, 188, doi 10.1007/s10661-016-5397-x)

Depending on the questions, objectives and motivation of the farmers, the appropriate species groups would be selected first and in the following step the indicators.

**>> We thank the reviewer for their suggestions. We have adjusted throughout the text to better explain the purpose of the study and the manuscript:**

**L94-104: “A key aspect of FRAMEwork was the development and testing of methods and tools for the monitoring and evaluation of farmland biodiversity, a main criterion for assessing the performance of the FCs. The overarching aim was to determine if biodiversity could be improved overall across the landscape by the FC selecting their own biodiversity targets and biodiversity-friendly farming initiatives. To test this, a Before-After-Control-Impact (BACI) experiment was designed, using a control for ten out of the eleven FCs.**

**The aim of this paper is to a) describe the FRAMEwork project’s methods to select and survey landscape-scale biodiversity indicators, and b) evaluate the appropriateness of the selected taxonomic indicators and the methodologies for data collection. However, the described protocols are also intended to be applicable for any future FC facilitators or practitioners to monitor their FC biodiversity from inception.”**

**>> Each FC selected their own biodiversity initiatives, and not many followed specific government-prescribed AES options, they were often more species-targeted and tailored to the needs of the FC. Considering this, we have tried to make the selection of the biodiversity indicators clearer in the text:**

**L142-149: “Identifying biodiversity indicators that communicated information regarding the status of the environment across the FC landscapes was an important step in the FRAMEwork project. These biodiversity indicators were not necessarily the target species/taxa of all the FCs, but it was intended that they would be proxies by which the overall biodiversity of each FC could be assessed over the course of the project. An initial list of indicators was drawn up by the project partners through an extensive literature review, these were then narrowed down through a rigorous evaluation process and constant feedback loop with project partners, facilitators, and biodiversity experts in a step-by-step elimination process to determine the final indicators for the project.”**

l. 205-213: Instead of describing single situations, please focus on the selection process of the biodiversity indicators and name the criteria for the single selection steps.

**>> The text has now been updated to include the step-by-step process we used to identify biodiversity indicators, so that the process is clearer and that others can emulate it with their own projects. The specific text mentioned above has been removed.**

**L146-167: “An initial list of indicators was drawn up by the project partners through an extensive literature review, these were then narrowed down through a rigorous evaluation process and constant feedback loop with project partners, facilitators, and biodiversity experts in a step-by-step elimination process to determine the final indicators for the project. Step 1: many indicators were identified from similar large-scale farmland biodiversity and pan-European projects such as Biodiversity indicators for organic and low-input farming systems (BioBio; (22)), Farm4Bio (23), Quantification of Ecosystem Services for Sustainable Agriculture (QuESSA; (24)), and European Monitoring of Biodiversity in Agricultural Landscapes (EMBAL;(18)), as well as additional indicators that were of interest to project partners (full list in S2 Appendix). The data collection methods and associated difficulty levels for monitoring each were also noted (S2 Appendix). Step 2: each indicator was evaluated for its relevance to the FRAMEwork project before being selected for the next stage i.e. its ability to be a proxy for biodiversity and inform at the FC landscape-scale. Step 3: all indicators were then assessed for their interest to multiple project partners (together with those not selected in step 2), including if project partners already had skills in identification or monitoring of the indicator, and brought forward to the next step. Step 4: it was agreed that a maximum of six indicators representing multiple categories (Kingdoms/Classes) would both suffice and be achievable within the timeframe for data collection, and the final list was defined, including the taxonomic rank to which all FCs would conduct monitoring. This resulting list of six biodiversity indicators to be monitored across all FCs included: flowering plants (to genus) and other vegetation features (including groups of species, percentage cover, colour diversity of flowers etc.); butterflies (identified to species level); bumblebees (to species or morphospecies level); solitary bees (to family, or genus and species where skills allowed); hoverflies (at the group level); and farmland birds (to species level).”**

l. 237-287: I strongly recommend shortening this section, as this is common information about the indicator functions of the selected taxonomic groups.

**>> Thank you for your suggestion. We have now adjusted this text and moved the adjusted text to the discussion as part of the evaluation of our methods.**

Surveys

Is it not relevant for the FC to receive timely answers whether the implemented biodiversity measures are having an effect?

**>> Thank you for your comment. Yes, it would be ideal to give the farmers feedback on their activities. However, it will likely be difficult to detect changes to biodiversity straight away, and attribute them directly to specific actions. Due to the timing constraints of the project, we hope to give what feedback we can at the end of the project.**

Why haven't innovative and non-lethal sampling/recording methods been chosen, such as for example the automatic recording and identification of moths (see moth monitoring with farmers in the Netherlands and Ireland; https://ddec1-0-en-ctp.trendmicro.com:443/wis/clicktime/v1/query?url=https%3a%2f%2fbiodiversityireland.ie%2fprojects%2ffarmer%2dmoth%2dmonitoring%2dproject%2f%3b&umid=84a5269a-61f9-4cce-b6fb-db024a79e04f&auth=07ed016851b7842c46c189839cce8de1b288bb7c-96edce0348b079266fdd7a9f0c465a05d6e1de18 https://assets.vlinderstichting.nl/docs/3c4b8794-5b31-4aa2-bcfb-b2542cd8c171.pdf) (it is also more cost-effective) or automated camera traps for insect diversity (R van Klink, T August, Y Bas, P Bodesheim, A Bonn, F Fossøy, TT Høye, E Jongejans, MHM Menz, A Miraldo, T Roslin, HE Roy, I Ruczyński, D Schigel, L Schäffler, JK Sheard, C Svinningsen, GF Tschan, J Wäldchen, VMA Zizka, J Åström, DE Bowler (2022) Emerging technologies revolutionize insect ecology and monitoring (2022) Trends in Ecology and Evolution 37:872-885) (in addition to bumblebee monitoring)? This could also circumvent the weaknesses of pantraps (l. 448-457).

**>> Thank you for your suggestion. At the start of the project, many of these technologies were too expensive to include. We have added the following line to the text to incorporate the knowledge of these technologies:**

**L519-521: “There are a few non-lethal technologies available (e.g. the DIOPSIS camera trap; (91), but most are emerging or highly expensive, which would make them impractical to implement in all the FCs of the project or expect future FCs to finance them.”**

Is there no chapter on data analysis?

**>> Because this is a methods paper and includes no real data or results, there is no data analysis section. We have included multiple brief explanations of different potential analyses that can be conducted in the ‘Anticipated results’ section.**

Results

What are the results of the biodiversity monitoring? Various species groups were recorded in the field, but why weren't the data presented here?

Unfortunately, the results section is not clear to me. It would certainly be good to describe and present the results in more detail way and establish a clear link to the methods section.

**>> This paper is just intended as a methods description and assessment of our protocols. The full data analysis which includes the results of the BACI design will be published at a later date. Instead, we now have a section called ‘Anticipated results’.**

Discussion

Unfortunately, the discussion is very brief and general. It would be desirable to discuss the singularity of the study in the context of result-based payment AES. However, I doubt that the sampling methods for insects and the numerous indicators listed are suitable for making statements on the effectiveness of measures within a CAP period, including interim evaluations.

**>> Thank you for your suggestion. The discussion has been completely reworked to include a critique of the biodiversity indicators and survey methods used. Additionally, the following lines have been added to the discussion in reference to the results-based payments:**

**L576-582: “Furthermore, there is a gradual shift towards AES payments becoming ‘results-based’ (RPBS; (66)). These schemes often require collaboration across the landscape to see true species recovery (101). Not only do FCs provide collaboration at the landscape-scale of the FC, but the above-described additional biodiversity indicators show the ease at which a targeted RBPS species could be integrated into the biodiversity monitoring template. This would result in standardised monitoring of the target species, ensuring that the biodiversity levels required to meet the expectations for payment are recorded.”**

Reviewer #2: General comments

The general concept described in this paper is excellent and extremely valuable: practical methods for coordinated landscape-scale conservation management across multiple landowners, critically with integrated monitoring that will allow evaluation of specific activities and feedback into management design to maximise long-term efficacy. It is important that this concept is promoted and encouraged widely, and the examples applying it to diverse landscapes across Europe are welcome.

**>> Thank you very much for your positive comments.**

Where I think the paper is limited and flawed is in (a) the application of protocols that are, largely, taken from national monitoring schemes and intended for national-scale inference to the local scale, where they are not reliable for delivery of repeatable data that will provide good evidence about the effects of management,

**>> Thank you for your comment. Because each cluster initiated their own, tailored, biodiversity-friendly activities throughout the 5 years, it wouldn’t have been possible to monitor all managements, or easily adjust analysis for the fact that new activities were added throughout the project. The aim in our methodology was to find an easy and reliable method to conduct across all FCs to capture overall biodiversity of a few specific taxa, rather than the specific managements put in place.**

and (b) the requirement for off-site, matched counterfactual areas, which are unlikely to be sustainable in the long term, even if suitable (unbiased) locations can be found, which is not a given.

**>> Thank you for highlighting this. The control sites were purely for the BACI design study. We do not envision any future FCs also contacting farmers and setting up control sites. If future projects wish to look at the impact FCs have, we have described what was important about the control sites:**

**L117-123: “It was hypothesised that the targeted biodiversity-friendly farming activities, initiated collaboratively by the FC, would lead to measurable improvements in overall farmland biodiversity which would be captured through standardised monitoring. Therefore, to determine the effect of FCs on overall farmland biodiversity, a BACI experiment was designed. Each FC was paired with a comparable control in a nearby, similar landscape and farming system (approximately 2 km away; Fig 1B). This approach allows the assessment of change in biodiversity over time by comparing trends in intervention and control sites before and after FC biodiversity measures were implemented.”**

I also think too much attention is paid to standardising approaches across landscapes that are not comparable anyway, but that is a minor issue.

**>> Thank you for your comment. The emphasis on standardisation was to make it easy for any European country to set up and begin monitoring a farmer cluster’s biodiversity. This manuscript describes the approach in a user friendly / easy to follow protocol, the protocols are applicable to biodiversity groups sensitive to environmental change present across Europe in multiple landscapes, the protocols are flexible enough to tailor to each landscape specifically as necessary.**

Another particular general issue with clusters is whether or how much they improve management performance over and above the same management done via individual, unconnected, farm-level AES agreements. There is sound reasoning to support it, but the point is that an assessment of the value in investing in cluster organisation needs to be done by comparing against the right thing, i.e. equivalent, unrelated agreements. However, it's also obviously of interest how well cluster farms benefit biodiversity relative to the unmanaged context. Monitoring should ideally allow both kinds of inference and, in the context of the monitoring approaches proposed here, it should be specified whether one or both are in scope.

**>> Thank you for your suggestion. As we understand it, the reviewer suggests that for this to be a sound study we would have engaged with 2 groups of farmers in each country/region and with the same farming system. But instead of the control we used, we would have had a control that conducted the same biodiversity sensitive farming practices. This would tell us if it the active participation of being in a FC, or simply the specific activities being undertaken, were driving any biodiversity changes. Although this is sound reasoning for a scientific study, it would not have been possible. 1) Engaging with farmers throughout Europe was very difficult and required a lot of troubleshooting, training, and incentives, and so having 2 groups of actively engaging farmers (in the project) in each country, would not have been achievable. 2) The farmers in the FC chose their own biodiversity sensitive actions. These were not necessarily subsidised AES, and not all farmers did all activities across their land. Additionally new activities were started throughout the time of the project. Therefore, we would have had to encourage the control group of farmers to also take up the same activities at the same times across the same proportions of their land, and not allow them to do any other activities outside of these. This would have been highly impractical and enforcing it would have been impossible. Therefore, we chose to approach farmers that would be considered as the control areas, and request access to conduct biodiversity surveys on their land, allowing them to conduct business as usual on their land, whatever that might have been.**

Overall, I am somewhat conflicted by the paper: I think the spirit of it is excellent and valuable, and the bottom-up organisation of farmers into collectives is a great approach, but the monitoring approaches could be improved significantly and I would not recommend them as a blueprint. Perhaps revision to present what is described here as one option, with an honest evaluation of the pros and cons, and description of alternatives, would be a way forward.

**>> We thank the reviewer for their positive comments, and would like to add that we have now done as they suggested. We do not suggest these as an overall blueprint for biodiversity monitoring across Europe, but as a useful tool for a FC facilitator to initiate. We explain how to adjust the survey squares, how to place the transects and adjust the lengths, and ideal timings for key landscape-scale taxa. We have made this clearer in the text (examples throughout this document) and have now done as the reviewer suggests and made an evaluation of the methods used, and described alternative methods in the discussion.**

I'd also suggest that the section describing hypothetical data could be dropped - I don't think it adds anything useful and does not prove anything without, at least, a formal simulation of a data set with realistic random variation.

**>> We would like to thank the reviewer for their suggestion. This has now been removed and an ‘anticipated results’ section has been added instead.**

Finally, I have various specific comments, as described below, including more detail on some of the points above.

Specific comments

Line 39. Should be "well-documented"

**>> corrected**

Line71-72. This is not true in England, and most jurisdictions, because it is a policy requirement, but the monitoring and evaluation is at the whole-scheme or national scale. It's arguable and variable how effective this is, but I think the point here is about monitoring at the agreement or local scale, which is not common or mandated.

**>> see below comment**

Line73-74. I think this is categorically untrue: in England at least, there is a lot of monitoring and evaluation work at the scheme and option levels, and assessment of national effect using national monitoring data. There are taxonomic biases, yes, and it can always be argued that studies could be done differently, but the text here paints a picture of a gulf that absolutely does not exist. There is also explicit feedback to scheme operation via Natural England. There is an issue, however, with time lags in getting reports into the public domain and the peer-reviewed literature, so the results may not be as well-known as would be ideal.

**>> Thank you for your comment. We have now adjusted the text in the introduction to:**

**L63-77: “It is generally unclear how AES are performing. Although some AESs are formally monitored by national governing bodies (e.g. in England by Natural England; (14)), and there is the capacity to monitor the multi-taxa success of AESs across the landscape through government-funded projects (15,16), there is no Europe-wide standardised biodiversity monitoring protocol that takes the implementation of biodiversity-friendly activities, such as AESs, into consideration. Moreover, individual farmers are not provided with feedback on the wider successes or failures of the actions they have instigated for each AES option, unless they hire an environmental advisor to conduct individual assessments and monitoring. Additionally, due to the way in which a large proportion of AESs have typically been taken up, by individual farm businesses as per the application process (though applications are submitted jointly in the Netherlands (17)), the potential to truly alter the landscape through coordination is missing. Thus, not only is it unclear if individually placed AES options are achieving their aims, but there is also no system in place to amend and improve an individual AES in situ on a farm, and there is little-to-no communication between farms on what actions have been put in place and where. This is where Farmer Clusters (FCs) have the potential to improve the current system.”**

Line78-79. I think this overstates the case - yes, most research has been at farm or field scale, but there has been landscape-scale work (not referenced here) and the importance of landscapes is well-known. Moreover, the fundamental reason for farm-level fragmentation is that the changes in practices that make up AES agreements are applied by individual farm businesses, which are independent agronomic units. Clearly biodiversity is not divided in the same way, which is why clusters are a good idea, but they are difficult or need specific effort because of the economics. This is perfectly adequate justification for this study without needing broad attacks on schemes and AES research.

**>> This line has been removed and see new text in above response.**

Line121-122. This is not true - for example, see Staley et al. 2021 (Staley, J. T., Redhead, J. W., O'Connor, R. S., Jarvis, S. G., Siriwardena, G. M., Henderson, I. G., ... & Pywell, R. F. (2021). Designing a survey to monitor multi-scale impacts of agri-environment schemes on mobile taxa. Journal of Environmental Management, 290, 112589). It's true, however, that effective monitoring of this kind needs a reasonably long time period before reporting, so results can easily take more than a decade before they are in the peer-reviewed public domain. Nevertheless, suggesting that no-one else is doing this kind of thing is inaccurate.

**>> Thank you for your comment. We have now updated the text in the introduction to:**

**L87-91: “Although several large multi-taxa programmes exist with well-developed protocols aimed at monitoring biodiversity (15,16,18), none were designed specifically to operate at the scale of the FC or the diverse landscape sizes that they encompass. There was a need to identify indicator species that were relevant to the landscape and aligned with the interests of the FCs, as well as a protocol adaptable across different European countries and farming systems.”**

Line129-131. This seems a laudable goal, but (a) the text above is heavily biased towards England and does not develop the policy-relevant needs in other countries, (b) while standardisation is a good idea, there is not much value in comparing data from different countries, relative to comparing with other places in the same country, i.e. with the same wildlife communities. Hence, matching to other national monitoring approaches seems a better idea.

**>> We thank the reviewer for their approval of our overall goal, and we have some responses to their concerns. A) We have now removed all text relating to forming a farmer cluster and the relevant policy requirements from this paper. B) The emphasis on standardisation was to make it easy for any European country to set up and begin monitoring a farmer cluster’s biodiversity, and although we have presented these methods as a single coherent protocol, we acknowledge and reference the use of national monitoring schemes that have been adopted throughout Europe regularly throughout the paper. We also highlight that these methods were for use in the project, and that any future farmer clusters could indeed simply follow their national monitoring schemes instead. However, if no schemes are present or they want something more complex, then we have provided the methodology in this paper, with all supporting files and documents they would need.**

**Example: L217-218: “The breeding bird survey used a transect walk method with the option to alternatively conduct point counts if these form part of the country’s national breeding bird survey.”**

Line140-141. Again re: standardisation, why try to collect standard data from diverse contexts? Surely it's better to target data collection on what works best for a given landscape?

**>> As above comment: The emphasis on standardisation was to make it easy for any European country to set up and begin monitoring a farmer cluster’s biodiversity. This manuscript describes the approach in a user friendly / easy to follow protocol, the protocols are applicable to biodiversity groups sensitive to environmental change present across Europe in multiple landscapes, the protocols are flexible enough to tailor to each landscape specifically as necessary. We have addressed the comment by adding explanation multiple times throughout the manuscript:**

**L180-182: “To ensure that biodiversity monitoring was representative of the FC landscape, a spatially balanced design was used. Each FC (and its control) was divided into squares scaled towards its average farm size, and surveys were conducted in a stratified sampling manner.”**

**L194-196: “By collecting data in this stratified format, comparable community data was collected for each biodiversity indicator within each FC.”**

**L256-257: “using well-known methodologies already in practice across several countries in Europe which have been proposed for the EU Pollinator Monitoring Scheme”**

**L354-356: “The protocols were based on similar projects whose methods were shown to be effective and could be easily implemented across all FCs.”**

**L380-382: “The above-described biodiversity monitoring protocols allowed for the collection of a large amount of quantitative data, working within a broad range of farming systems, landscapes, and habitat types across Europe.”**

**L382-383: “the stratified sampling method (survey squares) captured both the habitats (BHCs) and biodiversity across 103.75 km2 of land.”**

**L435-441: “To this end, FRAMEwork has adapted and tested well established biodiversity monitoring protocols, creating a novel, comprehensive and replicable process that aims to track biodiversity changes across a variety of farming systems, specifically at a multi-farm scale. The protocols presented here provide a practitioner-focused tool that can generate consistent and comparable biodiversity data in new and established FCs. They have undergone multiple rounds of refinement to not only improve the detail of the protocol, but to make them fully applicable in a range of countries, landscape types and farming systems across Europe.”**

**L461-466: “the Zeeasterweg, Lelystad FC in the Netherlands conducted a point count for each transect/survey square to align more closely with their country’s national monitoring scheme (54). Additionally, the Bass-Durance FC in France began their breeding bird surveys earlier than the protocol specified to capture migratory species as per their national bird survey, illustrating the flexibility of the overall approach and its ability to be adapted to satisfy local requirements.”**

**L566-570: “While the current suite of biodiversity indicators described above are recommended as a practical and ecologically meaningful starting point, the inclusion of additional species groups is encouraged where relevant and feasible.**

**The FRAMEwork survey square and transect placement protocol provides a template to monitor additional biodiversity indicators.”**

Line168-173. A clear weakness here is that the management programmes need to be long-term (at least 10 years). How are controls maintained for that length of time? How is it guaranteed that they do not join the cluster or independently adopt the same sort of management?

**>> Yes, we agree with the reviewer that in an ideal world we would have had over 10 years to see the full changes in biodiversity, but that due to the constraints of the project and it’s timing/funding, we could only test a 3-4 years comparison. We would hope that any future Farmer Cluster now has the means to formally monitor their biodiversity every 1-5 years over a long period of time. And again, there is no intention of future farmer clusters requiring a control, this is just for the BACI design of our experiment that is better detailed in the introduction and methods now.**

Line186-188. Again re: standardisation: why standardise if monitoring is for different purposes? Better to target methods to the desired outcomes?

**>> We thank the reviewer for their suggestion. We could have measured outcomes of certain practices, but what we wanted to know was if the overall biodiversity increased in a farmer cluster because of these activities, and the text change in the introduction to make this clearer has already been mentioned in an above response.**

Line188. It's not specified in Table 2 which regions targeted these species: presumably RLP is a not a target in the UK regions, as it's an introduced exotic species there?

**>> This sentence has been made clearer:**

**L112-116: “Each FC focused on its own environmental priorities, which ranged from improving the overall diversity of plants, birds, and pollinators, to actions such as planting hedgerows and protecting soil health, and even conserving specific species such as the red-legged partridge (Alectoris rufa) and barn owl (Tyto alba) (Nichols et al., unpublished).”**

Line260. "taxa" should be "taxon"

**>> Sentence no-longer in the text**

Line277-278. This is partly true, but depends on how exactly the hoverfly data are processed - for example, a species richness measure conflating adult counts of species that include larval aphid-eaters and others won't be very tightly linked to the actual predation service.

**>> This section has now been updated to:**

**L491-496: “Adult hoverflies are considered generalist foragers, with species typically visiting a wide range of flower species (85) (though see (86,87) for evidence of specialisation). Furthermore, the larvae of certain species predate on aphids, and can contribute to natural pest control as part of an Integrated Pest Management strategy (88,89). Therefore, the inclusion of hoverflies as a biodiversity indicator allows for the consideration of biological control in the landscape.”**

Line288-290. Noting again comments above about standardisation, the other key point here is that it is not just the broad groups being monitored that are important, it's how the data collected are processed into metrics for reporting and how they consider abundance, diversity, ecosystem service provision, etc.. It's quite possible to collect great survey data on a taxon but to lose any relationships with the real outcomes of interest in the analysis process. Discussion of this element is missing here.

**>> Thank you for your comments. In the original draft we had a “case study” example that showed what the data would look like (abundance, species richness, diversity data) and how we would use it. However, we have removed this and instead include a new ‘Anticipated results’ section, where we refer to the results as a “metric”. This is because we do not know the exact data analysis methods that will be used for each type of analysis and by each cluster and project partner. However, we have provided text of expected analysis.**

**L193-197: “By collecting data in this stratified format, comparable community data was collected for each biodiversity indicator within each FC. Furthermore, the data collected at co-locations within defined habitat types in each survey square allowed the relationships between vegetation and pollinator diversity metrics to be assessed, providing further insight.”**

**L400-418: “In terms of the BACI analysis, if the null hypothesis is rejected, the FCs will show an increase in biodiversity over time in comparison to the control sites (illustrated by Fig 4A). Conducting analysis for each biodiversity indicator and each FC separately allows identification of which indicators and FCs performed the best. This opens a discussion of why certain FCs or indicators have seen improvements, and whether the FC approach could be considered for improving overall farmland biodiversity across Europe. Additionally, the vegetation diversity metric and its relationship with the pollinator diversity metric will likely vary between habitat types (BHC) and can be assessed through a linear relationship for each FC. For example, floral vegetation may have the highest vegetation diversity metric and the strongest positive relationship with the pollinator diversity metric (Fig. 4B). This will allow for a discussion of which habitats and biodiversity sensitive farming actions might have the most positive impact on vegetation and pollinator biodiversity (e.g. reduced pesticide use, sowing of wildflower strips), and how this can impact overall farmland biodiversity. Furthermore, community dissimilarity analysis will determine the differences in species communities between the baseline year (before) and final year (after) of individual FCs (Fig 4C). If the null hypothesis is rejected and there is an increase of biodiversity in the FCs, the community of species found would change over time, and the ‘after’ community would be strongly associated with biodiversity (Fig. 4C). Finally, a before-after comparison of different biodiversity metrics in each FC will show which FCs saw the greatest improvements (Fig 4D). This allows discussion of why a certain FC might have seen these improvements.”**

Line304. "comparative" should be "comparable" or "similar"

**>> Corrected to comparable**

Line329-331. It's important to recognise that the BBS method and sampling approach are designed for inference about national trends, with a sample size of hundreds or thousands of squares per species. There is good evidence that smaller samples are sufficient for smaller regions with homogeneous landscapes, but the method was never intended and is not appropriate for inference at the level of individual squares or farms, so care (and justification) is needed to apply it in this way.

**>> Thank you for your comment. We have tried to make it clearer in the text throughout that we used the stratified squares to ensure the whole cluster (and control) landscape was represented, and each square had a single bird transect in it so that each square is representing the bird population. We have further explained in the comment below.**

Line 443. Should be "specimens"

**>> Corrected**

Line497. Presumably should be two bird transects?

**>> We have tried to make it clearer in the text throughout that we were simply using the BBS notations and monitoring criteria, not using the exact methods applied across the landscape at a national scale.**

**L217-224: “The breeding bird survey used a transect walk method with the option to alternatively conduct point counts if these form part of the country’s national breeding bird survey. The notation and monitoring criteria used were based on the protocol outlined in the British Trust for Ornithology’s (BTO) breeding bird survey (78). A single transect line of either 1 km or 500 m (for 500 m x 500 m survey squares) in length was identified in each survey square, running north-south or east-west, in a straight line where possible (Fig 2B & Fig 2C). Transects were ideally 500 m apart (and no closer than 200 m) to avoid double-counts. Depending on transect length, transects were divided into either ten or five 100 m sections and numbered 1-10 or 1-5 respectively.”**

**>> L456-457: “Because the FRAMEwork bird surveys were aligned to use the annotations from the BBS survey method, a transect line walk was opted for.”**

Line516 and pollinator/butterfly transect data processing. There is no mention in the MS of how the transect and pan trap data are to be/were combined. They are very different forms of data with different strengths and weaknesses, so need careful thought how they can be combined to inform about the same thing, especially with metrics using abundance.

**>> Thank you for your comment. The data will be considered separately, as the hoverflies as an indicator will be considered separately to the bumblebee and butterfly transect walk indicator data. Again, at this point we do not know the exact data analysis methods that will be used for each indicator or by each cluster and project partner. Here we are just presenting the anticipated results and potential avenues for analysis, as this paper focuses on the survey methods.**

Table 9. The design here, with respect to survey squares and numbers of visits, etc., is inspired by national schemes that aim to inform about the landscape scale and national trends, in which, for birds at least, minimum sample sizes of squares are at least 30-50 per species. It's true that smaller numbers would be fine in homogeneous landscapes, where the squares are likely to be good replicates of one another, but the sample sizes here are still generally too small, especially for monitoring species that are not found in all squares. I would suggest a greater density of survey transects in these smaller areas, probably with more visits, to produce data that will not be overly compromised by stochastic variation in detection.

**>> We would like to thank the reviewer for their comment. To confirm, we were not trying to capture species-specific trends, only gauge the overall bird diversity and community and it’s changes, ensuring enough test and control squares were selected (see S10 Appendix for the BACI calculation for the minimum number of squares to test). Furthermore, using only 1km^2^ squares, and sometimes 0.25 km^2^ squares, we only placed one transect per square to avoid double counts, as all squares and bird transects were typically surveyed on the same day for a single Cluster. Finally, as surveys were conducted for all 6 indictors throughout the spring-summer months, either by the facilitator or a paid for external contractor, there were only so many surveys that could be done within the timeframe and budgets of the project partners.**

Line 675. I don't follow where the stratification is in the design - stratified in respect of what?

**>> This line has been removed, and we hope we have clarified what we mean by stratified throughout this document.**

Line678. I don't agree and I think this is quite dangerous, actually: the methods applied for mobile taxa are drawn from national schemes and are designed for landscape-scale inference across hundreds of sites. With the stochasticity that is likely to affect the results of the sampling here, the data collected are not going to be reliable indicators for payment by results, which could have knock-on effects for the perceived value of the management being done in the eyes of land-managers and policymakers.

**>> This has been removed from the manuscript.**

Gavin Siriwardena, July 2024

________________________________________

6. PLOS authors have the option to publish the peer review history of their article (what does this mean?). If published, this will include your full peer review and any attached files.

If you choose “no”, your identity will remain anonymous but your review may still be made public.

Do you want your identity to be public for this peer review? For information about this choice, including consent withdrawal, please see our Privacy Policy.

Reviewer #1: No

Reviewer #2: Yes: Gavin Siriwardena

________________________________________
