# Supplementary material for: A standardised protocol for measuring farmland biodiversity outcomes across European Farmer Cluster landscapes
Source: PLoS One. 2026 Mar 25;21(3):e0345691. doi: 10.1371/journal.pone.0345691 (PMC13016360; doi:10.1371/journal.pone.0345691)
Supplement: Attachment — Submitted filename: Response_to_Reviewers_auresp_1.docx [file pone.0345691.s011.docx]

Journal Requirements:

When submitting your revision, we need you to address these additional requirements.

1. Please ensure that your manuscript meets PLOS ONE's style requirements, including those for file naming. The PLOS ONE style templates can be found at

<https://journals.plos.org/plosone/s/file?id=wjVg/PLOSOne_formatting_sample_main_body.pdf> and

<https://journals.plos.org/plosone/s/file?id=ba62/PLOSOne_formatting_sample_title_authors_affiliations.pdf>

**>> We have made edits to meet the style requirements.**

2. Thank you for stating the following financial disclosure:

“This Project has received funding from the European Union's Horizon 2020 research and innovation programme under grant agreement No 862731”

Please state what role the funders took in the study.  If the funders had no role, please state: "The funders had no role in study design, data collection and analysis, decision to publish, or preparation of the manuscript."

If this statement is not correct you must amend it as needed.

Please include this amended Role of Funder statement in your cover letter; we will change the online submission form on your behalf.

**>> We have included the updated statement in our cover letter.**

3. We note that you have referenced Nichols et al.” and “McHugh et al.” which has currently not yet been accepted for publication. Please remove this from your References and amend this to state in the body of your manuscript: “Nichols et al., unpublished” and “McHugh et al., unpublished” as detailed online in our guide for authors
<http://journals.plos.org/plosone/s/submission-guidelines#loc-reference-style>

**>> These papers are now published, so the reference list and citations have been updated to include them correctly.**

4. We note that Figures 1 and 2 in your submission contain map and satellite images which may be copyrighted. All PLOS content is published under the Creative Commons Attribution License (CC BY 4.0), which means that the manuscript, images, and Supporting Information files will be freely available online, and any third party is permitted to access, download, copy, distribute, and use these materials in any way, even commercially, with proper attribution.

For these reasons, we cannot publish previously copyrighted maps or satellite images created using proprietary data, such as Google software (Google Maps, Street View, and Earth). For more information, see our copyright guidelines: <http://journals.plos.org/plosone/s/licenses-and-copyright>.

We require you to either (1) present written permission from the copyright holder to publish these figures specifically under the CC BY 4.0 license, or (2) remove the figures from your submission:

1. You may seek permission from the original copyright holder of Figure(s) [#] to publish the content specifically under the CC BY 4.0 license.

We recommend that you contact the original copyright holder with the Content Permission Form (<http://journals.plos.org/plosone/s/file?id=7c09/content-permission-form.pdf>) and the following text:

“I request permission for the open-access journal PLOS ONE to publish XXX under the Creative Commons Attribution License (CCAL) CC BY 4.0 (<http://creativecommons.org/licenses/by/4.0/>). Please be aware that this license allows unrestricted use and distribution, even commercially, by third parties. Please reply and provide explicit written permission to publish XXX under a CC BY license and complete the attached form.”

Please upload the completed Content Permission Form or other proof of granted permissions as an "Other" file with your submission.

In the figure caption of the copyrighted figure, please include the following text: “Reprinted from [ref] under a CC BY license, with permission from [name of publisher], original copyright [original copyright year].”

2. If you are unable to obtain permission from the original copyright holder to publish these figures under the CC BY 4.0 license or if the copyright holder’s requirements are incompatible with the CC BY 4.0 license, please either i) remove the figure or ii) supply a replacement figure that complies with the CC BY 4.0 license. Please check copyright information on all replacement figures and update the figure caption with source information. If applicable, please specify in the figure caption text when a figure is similar but not identical to the original image and is therefore for illustrative purposes only.

The following resources for replacing copyrighted map figures may be helpful:

USGS National Map Viewer (public domain): <http://viewer.nationalmap.gov/viewer/>

The Gateway to Astronaut Photography of Earth (public domain): <http://eol.jsc.nasa.gov/sseop/clickmap/>

Maps at the CIA (public domain): <https://www.cia.gov/library/publications/the-world-factbook/index.html> and <https://www.cia.gov/library/publications/cia-maps-publications/index.html>

NASA Earth Observatory (public domain): <http://earthobservatory.nasa.gov/>

Landsat: <http://landsat.visibleearth.nasa.gov/>
USGS EROS (Earth Resources Observatory and Science (EROS) Center) (public domain): [http://eros.usgs.gov/#](http://eros.usgs.gov/)
Natural Earth (public domain): <http://www.naturalearthdata.com/>

**>> Both figures have now been revised, and contain maps that do not require copyright statements, or that have been correctly cited and can be used.**

5. We note that Figures 3 and s10 in your submission contain copyrighted images. All PLOS content is published under the Creative Commons Attribution License (CC BY 4.0), which means that the manuscript, images, and Supporting Information files will be freely available online, and any third party is permitted to access, download, copy, distribute, and use these materials in any way, even commercially, with proper attribution. For more information, see our copyright guidelines: <http://journals.plos.org/plosone/s/licenses-and-copyright>.

We require you to either (1) present written permission from the copyright holder to publish these figures specifically under the CC BY 4.0 license, or (2) remove the figures from your submission:

1. You may seek permission from the original copyright holder of Figure(s) [#] to publish the content specifically under the CC BY 4.0 license.

We recommend that you contact the original copyright holder with the Content Permission Form (<http://journals.plos.org/plosone/s/file?id=7c09/content-permission-form.pdf>) and the following text:

“I request permission for the open-access journal PLOS ONE to publish XXX under the Creative Commons Attribution License (CCAL) CC BY 4.0 (<http://creativecommons.org/licenses/by/4.0/>). Please be aware that this license allows unrestricted use and distribution, even commercially, by third parties. Please reply and provide explicit written permission to publish XXX under a CC BY license and complete the attached form.”

Please upload the completed Content Permission Form or other proof of granted permissions as an "Other" file with your submission.

In the figure caption of the copyrighted figure, please include the following text: “Reprinted from [ref] under a CC BY license, with permission from [name of publisher], original copyright [original copyright year].”

2. If you are unable to obtain permission from the original copyright holder to publish these figures under the CC BY 4.0 license or if the copyright holder’s requirements are incompatible with the CC BY 4.0 license, please either i) remove the figure or ii) supply a replacement figure that complies with the CC BY 4.0 license. Please check copyright information on all replacement figures and update the figure caption with source information. If applicable, please specify in the figure caption text when a figure is similar but not identical to the original image and is therefore for illustrative purposes only.

**>> We do not believe that any of the photographs used in Figure 3 are under any copyright laws. They were all take by project partner members and have been provided for use in this paper. If one of these has been highlighted as copyright, please tell us which one and where it is published, as we are unaware that they have been published elsewhere.**

**S10 has now been removed from the submission.**

6. If the reviewer comments include a recommendation to cite specific previously published works, please review and evaluate these publications to determine whether they are relevant and should be cited. There is no requirement to cite these works unless the editor has indicated otherwise. 

Additional Editor Comments:

Please respond to the concerns of the referees and revised the manuscript accordingly


[Note: HTML markup is below. Please do not edit.]

Reviewers' comments:

Reviewer's Responses to Questions

**Comments to the Author**

1. Is the manuscript technically sound, and do the data support the conclusions?

The manuscript must describe a technically sound piece of scientific research with data that supports the conclusions. Experiments must have been conducted rigorously, with appropriate controls, replication, and sample sizes. The conclusions must be drawn appropriately based on the data presented.

Reviewer #1: Yes

Reviewer #2: Partly

2. Has the statistical analysis been performed appropriately and rigorously?

Reviewer #1: N/A

Reviewer #2: I Don't Know

3. Have the authors made all data underlying the findings in their manuscript fully available?

The [PLOS Data policy](http://track.editorialmanager.com/CL0/http:%2F%2Fwww.plosone.org%2Fstatic%2Fpolicies.action%23sharing/1/010f019b58e6b078-ef184e9c-faf1-44fc-a2bc-506b2288fdaf-000000/OfoBvg_9Z8ky4AR9eYuXzh8OXpefujoqLfgHLbLLu3k=243) requires authors to make all data underlying the findings described in their manuscript fully available without restriction, with rare exception (please refer to the Data Availability Statement in the manuscript PDF file). The data should be provided as part of the manuscript or its supporting information, or deposited to a public repository. For example, in addition to summary statistics, the data points behind means, medians and variance measures should be available. If there are restrictions on publicly sharing data—e.g. participant privacy or use of data from a third party—those must be specified.

Reviewer #1: Yes

Reviewer #2: No

4. Is the manuscript presented in an intelligible fashion and written in standard English?

PLOS ONE does not copyedit accepted manuscripts, so the language in submitted articles must be clear, correct, and unambiguous. Any typographical or grammatical errors should be corrected at revision, so please note any specific errors here.

Reviewer #1: Yes

Reviewer #2: Yes

5. Review Comments to the Author

Please use the space provided to explain your answers to the questions above. You may also include additional comments for the author, including concerns about dual publication, research ethics, or publication ethics. (Please upload your review as an attachment if it exceeds 20,000 characters)

Reviewer #1: Comments on MS PONE-D-25-36303 entitled “A standardised protocol for measuring farmland biodiversity outcomes across European Farmer Cluster landscapes” by Nichols et al.

1. General comments:
This paper proposes a standardized protocol for monitoring farmland biodiversity in European Farmer Cluster landscapes. The protocol is designed to evaluate and unify methods for assessing biodiversity across different farming systems. It provides practical guidance on selecting survey squares and transects, as well as detailed procedures for surveying birds, pollinators, and vegetation. Comprising a suite of standardized sub-protocols, the framework is adaptable to diverse agricultural contexts. Finally, the authors evaluate the effectiveness of these biodiversity indicators and survey methods. This paper is of good scientific quality, thus may interest the readers of PLoS One. However, there are some aspects that could be improved for me. Therefore, I recommend this paper to have a major revision. After this major revision, the paper may contribute to the field.

**>> Thank you for your comments and providing suggestions for improvement.**

2. Specific comments
1) Is it necessary to include some indicators of biodiversity pressures such as the annual application of pesticides, annual application of fertilizers, per capita annual net income of rural households and so on. These indicators could facilitate the subsequent analysis after biodiversity investigation.
2) Is it necessary to include some indicators of ecosystem services such as water purification, reducing pesticide needs and so on.

3) Some biodiversity indicators at landscape scale could be derived from satellite remote sensing data such as forest cover, productivity and so on.

**>> Thank you for your suggestions. We agree that other environmental pressures are highly important, and were indeed considered. However, they were considered separately, as part of the Farmer Cluster sustainability analysis, and Farmland Ecosystem Assessment Support Tool (FEAST), so were not included in this methods protocol directly. We have added this text to the discussion:**

**L578-579: “Although other environmental indicators, services, and pressures were considered and are ecologically important…”**

**L588-594: “Additionally, environmental pressures such as pesticide use, fertiliser applications, water quality, and area of semi-natural habitat are all important indicators that were considered through a separate FC sustainability analysis (48), and ecosystem service indicators along with habitat suitability metrics were considered through the Farmland Ecosystem Assessment Support Tool (FEAST; (103)). These could be combined alongside the biodiversity indicators suggested above, should the FC have the funding and resources to include them.”**

4) This protocol aims to monitor biodiversity of farm cluster. The pro

**>> I emailed the editor to ask if the reviewer could send the full comment – I received the following text from the editor:**

**“The reviewer's comments are as follows:**

**The purpose of this protocol is to monitor biodiversity within farm clusters. It encompasses multiple taxon-specific subprotocols (e.g., for birds, insect pollinators, and vegetation). The current descriptions can be made more concise by increasing cross-references to these individual subprotocols.”**

**In response to this, we are struggling to see how the descriptions could be made more concise by cross-referencing. What the referee is proposing is unclear, and in the absence of an example, we don’t feel we can address this.**

5) I suggest to compare this protocol with similar protocols in discussion mentioned in the paper like biodiversity indicators for organic and low-input farming systems (BioBio), Farm4Bio, and European Monitoring of Biodiversity in Agricultural Landscapes.

**>> We have now added references of similar projects throughout the discussion, highlighting similarities and differences between our methods. Some examples:**

**L450-454: “The European Farmland Bird Indicator (EFBI) was created by Gregory et al. (51), and since then it has been used to predict that multiple European countries would witness a decline in farmland bird species in response to agricultural land-use change (52), making farmland birds a significant landscape-scale biodiversity indicator to monitor, hence their inclusion in many similar projects (16,24,25).”**

**L466-477: “Similar transect line surveys are already conducted as part of national bird census initiatives across Europe (56–58), with the data often used in large-scale biodiversity monitoring projects (20) and collated through the Pan-European Common Bird Monitoring Scheme, hence this method was chosen to be implemented in the FCs. However, some landscape-scale projects opt for different methods, such as “area searching” (mapping all birds that were seen or heard; (25)) or point counts, the latter with the potential to be used in place of transect walk. For example, the Zeeasterweg, Lelystad FC in the Netherlands conducted a point count for each transect/survey square to align more closely with their country’s national monitoring scheme (59). Additionally, the Bass-Durance FC in France began their breeding bird surveys earlier than the protocol specified to capture migratory species as per their national bird survey, illustrating the flexibility of the overall approach and its ability to be adapted to satisfy local requirements. Future farmland bird surveys could be adjusted as described here to allow flexibility around the FC’s local conditions.”**

**L479-480: “Insect pollinators were an important group to consider, used as indicators in many similar projects (16,20,24,25) …”**

**L521-523: “Although butterfly transect walks are typically about 1 km in length (80,98), the FRAMEwork pollinator protocol originally trialled a 3km continuous transect to capture a representation of the pollinators in the survey squares, similar in length to other projects that used longer transects (20,24,25).”**

**L557- 559: “Recording plant structure and percentage cover is a common theme in large-scale biodiversity monitoring projects (19,20,24,25), and so was considered a highly important biodiversity indicator.”**

**L563-567: “The FRAMEwork vegetation surveys were primarily based on the EMBAL survey (19) using a transect walk (e.g. (25)), which as a European protocol should be readily recognisable by multiple project partners and future FCs across Europe. Although plant surveys of some similar projects are conducted in plots or quadrats (24,104), to better capture the vegetation within the pollinator transect, a 20m length transect was opted for.”**

Reviewer #2: This research holds significant value for both ecological science and practical agricultural conservation in Europe. It directly addresses the critical challenge of farmland biodiversity loss by developing and rigorously evaluating a standardized, scalable monitoring protocol. The core innovation lies in its application within the "Farmer Cluster" model—a bottom-up, collaborative conservation approach. By creating a robust methodology to assess biodiversity outcomes across these clusters, the study provides an essential tool for empirically testing whether community-led agricultural initiatives can effectively reverse biodiversity decline at a meaningful landscape scale. The use of a Before-After-Control-Impact (BACI) experimental design significantly strengthens the potential to attribute observed changes in biodiversity to the interventions themselves, moving beyond mere observation to causal inference.

**>> Thank you for your kind comments.**

However, I do not believe this is a research article. It strikes me more as a research proposal or a work guideline. This depends on the journal's positioning.

**>> This paper is intended as a ‘Methods paper’, as outlined in PLOS ONE’s submission guidelines. We believe that our paper meets the requirements as a method’s paper. We are producing a single comprehensive protocol that allows capture of multi-taxa diversity, describes adjustments to different landscapes to specifically cater to European Farmer Cluster landscapes, which has not previously been done. By publishing Open Access in PLOS ONE, the accessibility would allow multiple facilitators to take up these methods for recording biodiversity on Farmer Clusters.**

In terms of strengthening practical guidance and feasibility, greater consideration should be given to the following issues—although these may not necessarily need to be addressed within the scope of the present study, but rather as key priorities for the authors’ future follow-up research. From the perspective of the work itself, I think at least three additional points need to be considered in its actual implementation.
1. The monitoring methods, although well-documented, can be labor-intensive and require significant expertise, potentially limiting their long-term application outside well-funded research projects. The BACI design, while robust, also depends heavily on the availability of suitable control sites, which may be difficult to maintain over time due to changing land-use practices.

**>> Thank you for your comments. We realise that the methods are incredibly detailed. This was in part to ensure than anyone wishing to replicate the surveys would find no ambiguity in the protocols and be confident in their application. Furthermore, a scoring system was developed during the biodiversity indicator selection phase, which considered the time and expertise required to collect the data and identify species to a certain level (S2 Appendix). This was to ensure that the optimum methods were used considering the costs and time constraints of the project, and we believe to be acceptable should anyone wish to monitor farmland biodiversity to the same level. Please note that we do suggest and discuss adjustments that could be made to produce similar, yet less time/cost-heavy data (see other responses).**

**The BACI design was for use in the short term (5 year) experiment that we ran to compare Farmer Clusters and control areas. We do not anticipate any new or established Farmer Cluster purposefully looking for a control area, as the monitoring would be done on their land only. The text has now been updated to make this clearer:**

**L175-177: “It is anticipated that any new or established FCs looking to monitor their biodiversity would consider biodiversity changes over time, rather than also looking for a control site to conduct a BACI analysis.”**

2. Integrate adaptive elements into the monitoring framework, allowing limited customization to regional conditions without compromising comparability. Incorporating remote sensing or automated monitoring tools could reduce costs and labor demands, making the protocol more scalable.

**>> Thank you for your comments. We believe we have allowed for as much flexibility as possible, from varying survey square sizes to adjusting dates, types, and replications of surveys depending on the country, landscape and local ecology. Please see examples from the text below, some include new text to make these flexibilities clearer:**

**L181-184: “Each FC (and its control) was divided into squares scaled towards its average farm size, and surveys were conducted in a stratified sampling manner. Each square was typically 1 km^2^, but smaller squares of 0.25 km^2^ (500 m x 500 m) were used in some FCs where farms were small or fragmented.”**

**L466-477: “Similar transect line surveys are already conducted as part of national bird census initiatives across Europe (56–58), with the data often used in large-scale biodiversity monitoring projects (20) and collated through the Pan-European Common Bird Monitoring Scheme, hence this method was chosen to be implemented in the FCs. However, some landscape-scale projects opt for different methods, such as “area searching” (mapping all birds that were seen or heard; (25)) or point counts, the latter with the potential to be used in place of transect walk. For example, the Zeeasterweg, Lelystad FC in the Netherlands conducted a point count for each transect/survey square to align more closely with their country’s national monitoring scheme (59). Additionally, the Bass-Durance FC in France began their breeding bird surveys earlier than the protocol specified to capture migratory species as per their national bird survey, illustrating the flexibility of the overall approach and its ability to be adapted to satisfy local requirements. Future farmland bird surveys could be adjusted as described here to allow flexibility around the FC’s local conditions.”**

**L539-546: “Furthermore, a recent large-scale synthesis across European habitats (97) found that total wild bee abundance was a very strong predictor of wild bee species diversity across different habitats and sampling methods, while bumblebee abundance alone was a weaker proxy. These findings indicate that incorporating total wild bee abundance into our surveys, alongside bumblebee counts, could improve the robustness of our transect protocol and provide a more reliable proxy for community-level pollinator diversity. Therefore, if identifying insect pollinators to species level is outside the abilities or funding of a future biodiversity survey, collecting wild bee abundance data could be used as an alternative proxy.”**

**L570-573: “However, there were lots of components to the survey making it complex and time consuming, and future FCs could consider switching to a single survey conducted at peak flowering season (May-June depending on climate and local conditions, recommended in the EMBAL guidelines (19)) instead of two surveys to relieve the survey effort required.”**

**L595-601: “The FRAMEwork survey square and transect placement protocol provides a template to monitor additional biodiversity indicators. For example, pitfall traps (e.g. for beetles), light traps (e.g. for moths), or ink footprint tunnels (e.g. for small mammals/hedgehogs; (107,108)) at singular points within each habitat type (mimicking the pan trap layout), could be deployed. Alternatively, sweep nets along the transects already identified (e.g. for foliar insects; (109)); or camera traps (e.g. for mammals) and acoustic recording devices (e.g. for birds or bats; (110)) could be placed one per square or one per habitat type within each square.”**

**We agree that monitoring tools would aid in reducing the survey effort and person-hours required to conduct the surveys. We have updated the text to include a suggestion:**

**L456-458: “Alternatively, either using point-count locations along the transect and a bird species identification application (e.g. Merlin Bird ID App) could greatly reduce the expertise level required.”**

3. Stronger integration of socio-economic indicators—such as farmer engagement and management changes—would help link ecological outcomes more directly to decision-making processes within farmer clusters.

**>> Thank you for your suggestion. We agree that socio-economic factors are highly important, in particular, the direct link between management changes and biodiversity outcomes. However, we feel that they are outside of the scope of this specific paper, as here we are trying to solely focus on the farmland taxa and how best to capture their diversity. Socio-economic indicators were considered in other parts of the project, such as financial incentives and decision-making (Fritschle et al., 2025), and others are combined with biodiversity in a sustainability analysis (Nichols, 2025). We have added some text to the paper to inform the readers:**

**L420-423: “By linking the biodiversity outcomes with other factors such as farmer engagement, financial incentives (47), and management changes (48), this could provide a more in-depth understanding of how the FC is working and which socio-economic indicators are linked to improved farmland biodiversity across FCs.”**

6. PLOS authors have the option to publish the peer review history of their article ([what does this mean?](https://track.editorialmanager.com/CL0/https:%2F%2Fjournals.plos.org%2Fplosone%2Fs%2Feditorial-and-peer-review-process%23loc-peer-review-history/1/010f019b58e6b078-ef184e9c-faf1-44fc-a2bc-506b2288fdaf-000000/d_VH01SqEfoXkYalGasO3_3N2mH2lBKUd94qOoTamOk=243)). If published, this will include your full peer review and any attached files.


If you choose “no”, your identity will remain anonymous but your review may still be made public.


**Do you want your identity to be public for this peer review?** For information about this choice, including consent withdrawal, please see our [Privacy Policy](https://track.editorialmanager.com/CL0/https:%2F%2Fwww.plos.org%2Fprivacy-policy/1/010f019b58e6b078-ef184e9c-faf1-44fc-a2bc-506b2288fdaf-000000/zO3Fg5FMxwlf3abuVLllLHoolX9wqNGD5pNwHuHDEbc=243).

Reviewer #1: No

Reviewer #2: No

[NOTE: If reviewer comments were submitted as an attachment file, they will be attached to this email and accessible via the submission site. Please log into your account, locate the manuscript record, and check for the action link "View Attachments". If this link does not appear, there are no attachment files.]

To ensure your figures meet our technical requirements, please review our figure guidelines: <https://journals.plos.org/plosone/s/figures>

You may also use PLOS’s free figure tool, NAAS, to help you prepare publication quality figures: <https://journals.plos.org/plosone/s/figures#loc-tools-for-figure-preparation>.

NAAS will assess whether your figures meet our technical requirements by comparing each figure against our figure specifications.
